# Ureteral stent discomfort: Etiology and management

**DOI:** 10.4103/0970-1591.57910

**Published:** 2009

**Authors:** Ricardo Miyaoka, Manoj Monga

**Affiliations:** Department of Urologic Surgery, University of Minnesota, Minneapolis, MN, USA

**Keywords:** Etiology, management, symptoms, treatment, ureteral stent

## Abstract

**Objectives:**

To review the evidence-based literature on the causes, characteristics, and options to manage double J stent-related symptoms.

**Methods:**

We performed a Medline database assessment on papers that investigated the prevalence, mechanisms, risk factors, bothersome and management of double-J stent-related symptoms. Articles in English were reviewed and summarized.

**Results:**

Stent-related symptoms have a high prevalence and may affect over 80% of patients. They include irritative voiding symptoms including frequency, urgency, dysuria, incomplete emptying; flank and suprapubic pain; incontinence, and hematuria. Assessment tools are important to determine their intensity and allow for comparisons between different points in the timeline. The Urinary Stent Symptom Questionnaire (USSQ) is the most proper tool used for this purpose. Management should be focused on the prevention and management of symptoms. In this sense, research has focused on new materials and stent designs that would be more compatible to the physiologic properties of the urinary tract and medications that can ameliorate the sensitivity and motor response of the bladder.

**Conclusions:**

Stent-related symptoms are very common in the Urological clinical setting. It is of major importance for the urologist to understand their physiopathology and to be familiar with ways to avoid or manage them.

## INTRODUCTION

Since its first description in 1967 by Zimskind, *et al*[1] the double-J ureteral stent has been an indispensable tool in the urologist's surgical armamentarium.

By definition, the double-J or pigtail stent is a catheter or tube placed within the ureteral lumen in a retrograde or antegrade fashion in order to maintain its patency.[2] The pigtail catheter provides a self-retaining capability due to a double coil design at proximal and distal ends that work to securely anchor the stent in the upper urinary tract (renal pelvis and upper calyx) and the bladder. This prevents stent migration proximally or distally despite urinary flow, patient movement, and ureteral peristalsis.

Ureteral stents play a major role in a wide range of situations where urinary drainage is needed. Urgent indications include cases of obstructive pyelonephritis and intolerable acute renal colic;[3] safety indications following endoscopic procedures include ureteral edema or perforation, steinstrasse,[4] history of renal failure, and solitary or transplant kidney. Relative indications would still include stone burden larger than 2 cm undergoing extracorporeal shockwave lithotripsy, pregnancy, long-standing impacted stone, recent history of urinary tract infection or sepsis, stent to passive dilate the ureter and/or ureteral orifice, prolonged endoscopic operative time (over 45 minutes) and any patient with imminent post-operative plans such as a second-look ureteroscopy[5] [Table 1].

**Table 1:** Current indications for stent replacement

Urgent
Obstructive pyelonephritis
Intolerable acute renal colic
Renal failure secondary to ureteral obstruction
Safety related
Ureteral edema
Ureteral perforation
Steinstrasse
Previous history of renal failure
Solitary kidney
Transplant kidney
Relative
Stone burden >2 cm before SWL
Pregnancy
Long-standing impacted stone
Recent history of urinary tract infection or sepsis
Passive dilation of ureteral orifice and ureter
Prolonged endoscopic operative time
Patients with imminent post operative plans (2^nd^ look)

Despite these legitimate indications, ureteral stents are thought to be overused in contemporary urology practice. In a recently published survey among community and academic practicing urologists from worldwide centers, Auge *et al.* reported that 98% of the responders perform ureteroscopic stone surgery in their routine. Of these, two-thirds would place a stent more than 50% of the time and 13% would always place a post-operative stent, even though intolerance to the stent presence was the most significant problem addressed by patients (98%).[6] Despite the growing evidence of well-conducted randomized prospective trials demonstrating the safety of not leaving a post-ureteroscopy ureteral stent, many urologists still place stents after the majority of uncomplicated stone-removal procedure.[7–9]

## STENT-RELATED SYMPTOMS: EPIDEMIOLOGY AND ETIOLOGY

Stent discomfort can vary from one patient to another in an idiosyncratic manner, but is believed to affect over 80% of patients.[10,11] Conversely, a study by Hao *et al.* reported a low incidence of 19.6% of stent-related complications.[12] In this study, however, analysis was taken upon a cohort composed by patients who underwent different endourologic procedures percutaneous Nephrolithotomy, ureteroscopy, shockwave lithotrips (PCNL, URS, SWL) and the means of assessment are not clarified, which makes comparison less than reliable.

Several studies in literature describe the symptoms related to ureteral stents and their respective estimated incidence: irritative voiding symptoms including frequency (50-60%), urgency (57-60%), dysuria (40%), incomplete emptying (76%), flank (19-32%) and suprapubic pain (30%), incontinence, and hematuria (25%) are included.[3,8,10,12–16]

The mechanisms leading to the above mentioned symptoms have yet to be elucidated.

Frequency is attributed to a mechanical stimulus that comes from the bladder coil. Along with urgency, it affects a significant proportion of patients (60%). Daytime frequency distinguished by the lack of coexisting nocturia suggests that mechanical stimulation relates to physical activities and/or awareness of this stimulation during the day, which would not be significant during the night. Objective assessment through frequency volume charts corroborates this theory.[10] Recently, investigators confirmed that stent displacement with physical activity may impact stent discomfort. In a small study of 6 patients, they noted up to 2.5 cm of movement of the renal coil or bladder coil and associated bowing in the proximal ureter with alteration in patient position.[17]

Urgency is thought to be a direct result from the presence of the stent, which may also unmask or exacerbate pre-existing subclinical detrusor overactivity.[10]

Dysuria is usually experienced at the end of voiding. It has been proposed that dysuria is secondary to trigonal irritation by the distal end of the stent when it crosses the midline or forms an incomplete loop.[15] In a similar way, a recent published randomized clinical trial confirmed that urgency and dysuria were more common with longer stents and negatively impacted the patients' quality of life.[18]

Flank pain is most likely a result of urine reflux towards the kidney that leads to an excessive rise in intrapelvic pressure that ultimately translates into pain.[19,20] It is usually mild to moderate and is not influenced by the position of the proximal coil either in the upper calyx or in the renal pelvis.[18,21]

Suprapubic pain can result from local bladder irritation by the distal coil or as a secondary sign of associated complication such as encrustation or infection.[22]

Hematuria may result from surgical management of existing disease and from the stent placement itself as well.[21]

Incontinence typically occurs in association with episodes of urgency, the physiopathology of which was addressed above, or as a result of stent migration beyond the bladder neck into the proximal urethra bypassing the urethral sphincteric mechanism of continence.[23]

It should be emphasized that all symptoms can be a consequence of associated stent morbidities such as urinary tract infection and encrustation, and the presence of these should be excluded by urinalysis and imaging as indicated.[21]

## ASSESSMENT TOOLS

Joshi *et al.* reported on the first study to objectively evaluate the symptomatology associated with stents. They prospectively assessed the prevalence and bother of various urinary tract symptoms caused by indwelling ureteral catheters using validated questionnaires (International Prostatic Symptoms Score - IPSS, International Continence Society male questionnaire, Quality of Life questionnaires, and the Bristol Female Lower Urinary Tract Symptoms questionnaire - BFLUTS). Although they succeeded in showing the association of urinary symptoms with stents and their negative impact on patients' quality of life, the most important contribution was to bring to attention the need for the development of a stent-specific measuring tool.[10]

In order to better orient clinical decision making and practice, they later developed and validated a questionnaire to specifically address this purpose. The Ureteral Stent Symptom Questionnaire (USSQ) consists of 38 items examining 6 sections: pain, voiding symptoms, work performance, sexual matters, overall general health, and additional problems.[24] It was shown that 76% of patients had urinary symptoms, 70% had pain severe enough to reduce their activities by 50% and felt less healthy in general, and 32% experienced sexual dysfunction.[25,26]

The use of the USSQ has subsequently made it possible to classify symptoms related to specific stent manufacturers and to measure and compare their differences proving to be a sensitive tool. Lee, *et al.* compared 6F ureteral stents provided by 5 different manufacturers (Bard-Inlay, Cook Endo-Sof, Microvasive Contour, Applied Medical Vertex, and Surgitek Classic Double-Pigtail stent). Bard-Inlay was associated to less severe urinary symptoms although it had no impact on pain scores or narcotic use.[27]

The complete USSQ form is available at www.bui.ac.uk/endourology and www.endourology.org

## MANAGEMENT

Treatment strategies can be classified into 2 different approaches:

1. Prevention of stent-related symptoms
2. Management of stent-related symptoms

For the first category, accurate stent indications, pre-stenting maneuvers, and improvements in both stent design and structure have been the main focus. For the second, pharmacology has focused on minimizing the motor and sensory bladder response to stent presence. Lower Urinary Tract Symptoms

## PREVENTION

### Precise indications

“Preventing as the best treatment” approach is a universal concept that adequately applies to stent use in the Urology practice. Considering its significant morbidity, stents should be used under conscious and evidence-based criteria.

A prospective multi-institutional randomized study involving 113 patients who underwent uncomplicated semirigid ureteroscopy for the treatment of distal ureteral calculi demonstrated significantly more post-operative pain and Lower Urinary Tract Symptoms (LUTS) in stented patients *vs.* nonstented patients.[9] These findings are in accordance with a systematic review by Haleblian *et al.* who reported that routine stenting following an uncomplicated ureteroscopy does not lower the complication rates, unplanned hospital visits or rehospitalizations, increase treatment costs, and is ultimately not necessary, except in pregnant women. They also reported that ureteral stenting does not improve stone-free rates and suggested that stents should not be placed before SWL, except in a selected pediatric population patients with large stone burden.[8]

History of renal failure, solitary kidney and transplant kidney continue to be absolute indications for stent placement after uncomplicated ureteroscopy. Complicated ureteroscopy as defined by ureteral perforation is an indication for ureteral stenting. Relative indications are significant ureteral edema at the completion of the procedure, pregnancy, stone burden greater than 2 cm, longstanding impacted stone, and recent history of urinary tract infection or sepsis.[5]

### Pre-stenting maneuvers

Sur, *et al*. evaluated the role of periureteral anesthetic injection preceding stent insertion hoping to reduce urinary symptoms. They conducted a single-blinded, randomized study in 22 patients who received 50 mg of ropivacaine *vs*. saline injections submucosally around the ureteral orifice. Results failed to show significant changes in postoperative pain, voiding symptoms, or narcotic requirements. However, the authors conclude that the administration of an analgesic before the nociceptive input may reduce the need for postoperative analgesia (preemptive pain control theory) and suggest that further studies should be conducted with larger populations.[14]

### Stent length and positioning

Stent length seems to play a relevant role in stent-related symptoms since it is directly related to bladder irritation. Several different ways to assess the ideal stent length have been suggested.

Lee, *et al.*[27] used a reference table in which a corresponding stent length was selected for each given specific height range. Ho, *et al.* prospectively evaluated 87 patients and assessed their stent-related symptoms. They determined that a 22-cm stent would be more appropriate for those whose height ranges from 149.5 cm to 178.5 cm with a median of 161.9 cm.[21]

Mathematic formulas have also been proposed to calculate stent length. Hao, *et al.* used the following: (length = 0.125 × body height + 0.5 cm) or the vertical distance from the second lumbar vertebra to the pubic symphysis minus 2 cm.[12] Hruby, *et al.*[28] calculated that the xyphoid process to pubic symphysis distance as well as acromium process to the head of the ulna distance can both be used to predict double J length.

In the pediatric population, a rule of thumb has been proposed to determine the suitable JJ stent regardless of gender or size, which is simply to add 10 to the age of the patient (stent length=patient age [years] + 10).[29] [Table 2].

**Table 2:** Stent length calculation formulas

Author	Formula
Ho, *et al.*	22-cm stent for pts ranging from 149.5 - 178.5 cm
Hao, *et al.*	(Length=0.125 × body height + 0.5 cm), or the vertical distance from the second lumbar vertebra to the pubic symphysis minus 2 cm
Palmer, *et al.*	[stent length=patient age (years) + 10]

Regarding stent positioning, a stent that crosses the midline implies an overlong stent and should be avoided since it may be associated with more irritative bladder symptoms. The proximal loop positioning does not seem to correlate with flank pain and is likely to be associated with placement technique rather than stent length.[21]

### Stent coating

Encrustation of the stent may lead to ipsilateral ureteral obstruction and renal colic. Stents that are in situ for more than 12 weeks have a 76% incidence of encrustation.[30]

The ureteral stent provides a surface for biofilm formation, bacterial colonization, and encrustation. After biofilm formation, encased bacteria gain dormancy and resistance to eradication by antibiotic agents.[31] Therefore, research has been focusing on preventing this process.

In an attempt to incorporate drugs to the core of the polymeric structure of the stent material, a triclosan loaded stent was developed (Boston Scientific Triumph™) and was shown to decrease bacterial growth in artificially infected urine with *Proteus mirabilis*, probably by preventing bacterial adherence to the biofilm of the drug coated stent which in turn might lead to a decrease in stent encrustation.[32]

Drug-eluting stents using the anticancer drug Paclitaxel was tested in a porcine model, with the hope of reducing the hyperplastic reaction of the urothelium. A mild inflammatory reaction without hindering luminal patency was noted.[33]

Watterson, *et al*.[34] coated circular silicone disks with an oxalate-degrading enzyme and implanted them in a rabbit model for 30 days. Results showed a 21% and 40% reduction in the dry weight of encrustation and calcium within the encrustation, respectively, in the experimental group versus control group.

Riedl, *et al*.[35] compared heparin-coated polyurethane ureteral stents with uncoated stents during a 6-week period showing effective inhibition of both biofilm and encrustation formation by the first group. In a similar fashion, a study by Zupkas, *et al*.[36] used a rabbit bladder implantation model to compare silicon rings coated with pentosanpolysulfate, a semisynthetic polysaccharide chemically similar to heparin, with uncoated silicon rings. Pentosanpolysulfate coated rings resulted in an eight-fold reduction in encrustation formation.

In another study, Multanen, *et al*. demonstrated the advantages of a silver nitrate and ofloxacin-blended copolymer-coated urospiral stents over pure copolymer coating stents showing less tissue reaction and preventing biofilm and encrustation formation.[37]

Glycosaminoglycan-coated stents also have demonstrated increased resistance to encrustation in experimental studies.[38] Advances and changes in design have been made aiming to improve urinary stent-related symptoms.

A self-expandable mesh stent was developed in an attempt to preserve drainage while reducing irritative symptoms. Olweny, *et al*.[39] demonstrated less inflammatory tissue reaction and a tendency toward better urinary flow at 1 and 6 weeks when comparing this new prototype to a standard 7F double pigtail polyurethane stent in a porcine model, although results did not reach statistical significance.

A different design was introduced in the Tail Stent™ model (Microvasive Urology/ Boston Scientific) with the objective to minimize irritative bladder symptoms. This is a stent with a proximal 7F pigtail and a shaft that tapers to a lumenless straight 3F tail that lies in the bladder.[40] Tail stents were found to produce fewer irritative symptoms than standard 7F double J stents in a randomized single-blind trial involving 60 patients.[41]

Dual-durometer stents combine a firm biomaterial at the renal end which smoothly transitions to a soft biomaterial at the bladder end intended to reduce mechanical irritation of the vesical urothelium. Sof-Curl™ and the Polaris™ stents are the available models. They are coated with hydrophilic-bonded hydrogel that decreases their coefficients of friction. The Polaris™ showed lower flexural strength when compared with 5 other stents, which is believed to minimize bladder discomfort.[42]

Anatomical investigation has shown that in order to improve stent discomfort, future stent designs must take into account the range of motion of the ureter during changes in body position. It seems that stent movements are a combination of bowing in the proximal ureter and moving within the bladder.[17]

## PHARMACOLOGY

Currently, there is a wide variety of medical treatments to relieve irritative bladder symptoms. They can be directly instilled inside the bladder or taken orally.

Intravesical instillation of chemical agents aiming to improve stent discomfort is a relatively recent approach. Beiko, *et al*. conducted a double-blind prospective trial on 42 patients randomized to receive intravesical instillation of one of three chemicals (ketorolac, alkalinized lidocaine, or oxybutynin) versus saline, as control, immediately after stent placement at the time of SWL. No side effects were reported and ketorolac was associated to a significant decrease in irritative symptoms at 1-hour after intervention.[43] Subsequent studies failed to demonstrate differences between intravesical agents for relief of stent-related symptoms.

In 2006, a study by Deliveliotis, *et al*. investigated the role of alpha_1_-blockers for treating these symptoms. They performed a prospective, randomized, placebo-controlled study to compare the impact of stent symptoms on patients' Quality of life (QOL) using a validated questionnaire (USSQ). Patients who underwent cystoscopically placed stents to treat stone-related hydronephrosis were given 10 mg alfuzosin once daily for 4 weeks. Results showed a decrease in mean urinary symptom index (p<0.001), frequency of stent-related pain (p=0.027), and an improvement in the general health index score (p<0.001) for patients in the alfuzosin group.[44]

In a recently published study, Beddingfield *et al*. also evaluated alfuzosin as an adjunct to the improvement of stent related symptoms. A total of 55 patients were randomized to receive either 10 mg alfuzosin hydrochloride or placebo once a day for 10 days following post ureteroscopy stent placement. USSQ and narcotic use diary were assessed. Results showed a significant improvement for the alfuzosin group regarding sleep interrupted by pain, frequency of painkiller, pain interfering with life, and flank pain associated with micturition (p<0.005). The placebo group showed worsening for these same symptoms. Although alfuzosin led to a decrease in the frequency of narcotic use, the total amount was not changed.[45]

Another study compared alfuzosin with tolterodine ER and placebo.[46] A total of 52 patients were randomized after different endourological procedures and stent placement to receive one of the following three doses: 10 mg alfuzosin, 4 mg tolterodine ER, or placebo for a 6-week period. Both alfuzosin and tolterodine were able to improve pain and urinary symptom index scores when compared with placebo (p=0.02 and p=0.008, respectively).

Tamsulosin also proved to be efficacious in improving stent-related morbidity. In a study by Damiano, *et al.* it was shown to decrease flank pain and urinary symptoms at 1 week and increase the general health index score, although this study was not double-blinded or placebo controlled.[47]

In contrast to this data, Norris, *et al*. recently published their experience with a small but well conducted double-blind, placebo-controlled study comparing ER oxybutynin, phenazopyridine, and placebo in patients who had a stent place after ureteroscopy.[48] Assessment tools included a questionnaire for stent symptoms, visual analog scale scores, and requirement of narcotic medications. Results did not show differences for flank pain, suprapubic pain, urinary frequency, urgency, dysuria, narcotic usage, or hematuria (except for phenazopyridine versus placebo on Day 2).

## CONCLUSION

Studies directed to assess ureteral stent discomfort remain a challenge since pain is the primary end-point, which can lead to subjective and variable conclusions and may be influenced by confounding variables such as BMI, age, and comorbidities. Also, despite strict exclusion criteria, other aspects may be difficult to identify at the time of enrollment. Recurrent stone formers who suffer from chronic pain or narcotic dependency are examples. Besides, the pain provoked by the primary pathology or associated intervention such as ureteroscopy can confound the cause-effect analysis of the presence of the stent.

In the face of this, future efforts must focus on the refinement of assessment tools and continued development of stent materials targeting biocompatible/ biodegradable devices that would induce minimal tissue reaction and at the same time reliably degrade either on demand or in a predictable pattern.

Medications to decrease morbidity should be regarded as a palliative adjunctive approach, but seem to be a more reachable solution in the short-term.
